# The effects of fixed and removable orthodontic retainers: a systematic review

**DOI:** 10.1186/s40510-016-0137-x

**Published:** 2016-07-26

**Authors:** Dalya Al-Moghrabi, Nikolaos Pandis, Padhraig S Fleming

**Affiliations:** 1 Barts and The London School of Medicine and Dentistry, Queen Mary University of London, London, E1 2AD UK; 2 Dental School, Medical Faculty, University of Bern, Bern, Switzerland

**Keywords:** Orthodontic retainer, Periodontal, Survival rate, Failure rate, Cost-effectiveness, Patient-reported outcomes

## Abstract

**Objective:**

In the view of the widespread acceptance of indefinite retention, it is important to determine the effects of fixed and removable orthodontic retainers on periodontal health, survival and failure rates of retainers, cost-effectiveness, and impact of orthodontic retainers on patient-reported outcomes.

**Methods:**

A comprehensive literature search was undertaken based on a defined electronic and gray literature search strategy (PROSPERO: CRD42015029169). The following databases were searched (up to October 2015); MEDLINE via OVID, PubMed, the Cochrane Central Register of Controlled Trials, LILACS, BBO, ClinicalTrials.gov, the National Research Register, and ProQuest Dissertation and Thesis database. Randomized and non-randomized controlled clinical trials, prospective cohort studies, and case series (minimum sample size of 20) with minimum follow-up periods of 6 months reporting periodontal health, survival and failure rates of retainers, cost-effectiveness, and impact of orthodontic retainers on patient-reported outcomes were identified. The Cochrane Collaboration’s Risk of Bias tool and Newcastle-Ottawa Scale were used to assess the quality of included trials.

**Results:**

Twenty-four studies were identified, 18 randomized controlled trials and 6 prospective cohort studies. Of these, only 16 were deemed to be of high quality. Meta-analysis was unfeasible due to considerable clinical heterogeneity and variations in outcome measures. The mean failure risk for mandibular stainless steel fixed retainers bonded from canine to canine was 0.29 (95 % confidence interval [CI] 0.26, 0.33) and for those bonded to canines only was 0.25 (95 % CI: 0.16, 0.33). A meta-regression suggested that failure of fixed stainless steel mandibular retainers was not directly related to the period elapsed since placement (*P* = 0.938).

**Conclusion:**

Further well-designed prospective studies are needed to elucidate the benefits and potential harms associated with orthodontic retainers.

## Review

### Introduction

Retention procedures are considered necessary to maintain the corrected position of teeth following orthodontic treatment and to mitigate against characteristic age-related changes which, if unchecked, are known to culminate in mandibular anterior crowding [1]. Retention procedures are continually being refined with a recognition that existing protocols are infallible [2]. Nevertheless, both fixed and removable retainers continue to be in vogue, although adjunctive procedures including interproximal enamel reduction and minor oral surgical procedures have also been advocated.

A recent Cochrane review exposed a lack of high-quality evidence to favor one method of retention over another in terms of stability [3]. Given this absence of definitive evidence, retainer selection is often based on individual preference. This is evidenced by marked geographical variation with maxillary Hawley or vacuum-formed retainers and mandibular fixed lingual retainers with full-time wear of removable retainers most popular in the USA [4, 5]. In Australia and New Zealand, mandibular fixed and maxillary vacuum-formed retainers are shown to be the most prevalent combination [6], while a preference for the use of fixed retainers in both arches has been shown in the Netherlands [7].

The duration of wear of orthodontic retainers has long been a dilemma in orthodontics. However, there is now widespread acceptance of the necessity for indefinite retention to minimize both relapse and maturational changes [5, 8]. Prolonged retention may pose increased risk to the periodontium and dental hard tissues; it is therefore important to investigate the implications of the long-term use of fixed and removable retainers on the supporting tissues [3, 9, 10].

A further consideration is patient experiences of retention and compliance with prolonged retention regimes; it is intuitive to expect that co-operation with retention regimes would decline over time. Moreover, both fixed and removable retainers are prone to breakage, loss, and degradation [2, 11]. Repeated breakage and requirement for replacement may have implications for the cost-effectiveness of both fixed and removable approaches. There is however limited evidence concerning the cost-effectiveness of either approach [12, 13].

The primary aim of this systematic review was to determine the influence of fixed and removable orthodontic retainers on periodontal health in patients who have completed orthodontic treatment with fixed appliances. A secondary aim was to evaluate survival and failure rates, impact of orthodontic retainers on patient-reported outcomes, and cost-effectiveness.

### Materials and methods

This protocol for this systematic review was registered on PROSPERO (www.crd.york.ac.uk/prospero; CRD42015029169). The following selection criteria were applied:

- Study design: randomized and non-randomized controlled clinical trials, prospective cohort studies, and case series (with a minimum sample size of 20 patients) with minimum follow-up periods of 6 months
- Participants: patients having had orthodontic treatment with fixed or removable appliances followed by orthodontic retention
  - Interventions: fixed retainers, removable retainers, and interproximal reduction
  - Outcome measures: periodontal outcomes, survival and failure rates (including detachment of fixed retainers, breakages, retainer loss, or the need for replacement), patient-reported outcomes, and cost-effectiveness measures

### Search strategy for identification of studies

The following databases were searched up to October 2015 without language restrictions: MEDLINE via OVID (Appendix 1), PubMed, the Cochrane Central Register of Controlled Trials (CENTRAL), and LILACS and BBO databases. Unpublished trials were searched electronically using ClinicalTrials.gov (www.clinicaltrials.gov), the National Research Register (www.controlled-trials.com), and ProQuest Dissertation and Thesis database (http://pqdtopen.proquest.com).

### Assessment of relevance, validity, and data extraction

Full texts of relevant abstracts were retrieved. Data was tabulated using pre-piloted data collection forms by two authors (DA, PSF). Data extracted included: (1) study design; (2) sample: size, demographics, and clinical characteristics; (3) intervention: fixed appliances, removable appliances, or interproximal reduction; (4) follow-up period; (5) maxillary/mandibular arch; and (6) outcomes (primary and secondary).

### Risk of bias (quality) assessment

For randomized controlled trials sequence generation, allocation concealment, blinding of outcome assessors, incomplete outcome data, selective reporting, and other biases were assessed using the Cochrane Collaboration’s Risk of Bias tool. Any disagreement was resolved by joint discussion (DA, PSF). Only studies at low or unclear risk of bias overall were to be included in the meta-analysis. The methodological quality of the included non-randomized studies was assessed using the Newcastle-Ottawa Scale. Studies adjudged to be of moderate or high methodological quality overall (more than five stars) were to be included in the meta-analysis. The authors of the included studies were contacted for clarification if required.

### Strategy for data synthesis

Clinical heterogeneity was assessed according to the treatment interventions, wear regimen for removable retainers, measurement approach, and location of the retainers. For periodontal outcomes, the index used and surfaces examined were considered. Statistical heterogeneity was to be assessed by inspecting a graphic display of the estimated treatment effects from individual trials with associated 95 % confidence intervals. Heterogeneity would be quantified using *I*-squared with values above 50 % indicative of moderate to high heterogeneity which might preclude meta-analysis. A weighted treatment effect was to be calculated, and the results for retainer failure were expressed as odds ratios. All statistical analyses were undertaken using the Stata statistical software package (version 12.1; StataCorp, College Station, Tex).

### Results

#### Description of the included studies

**Fig. 1:**
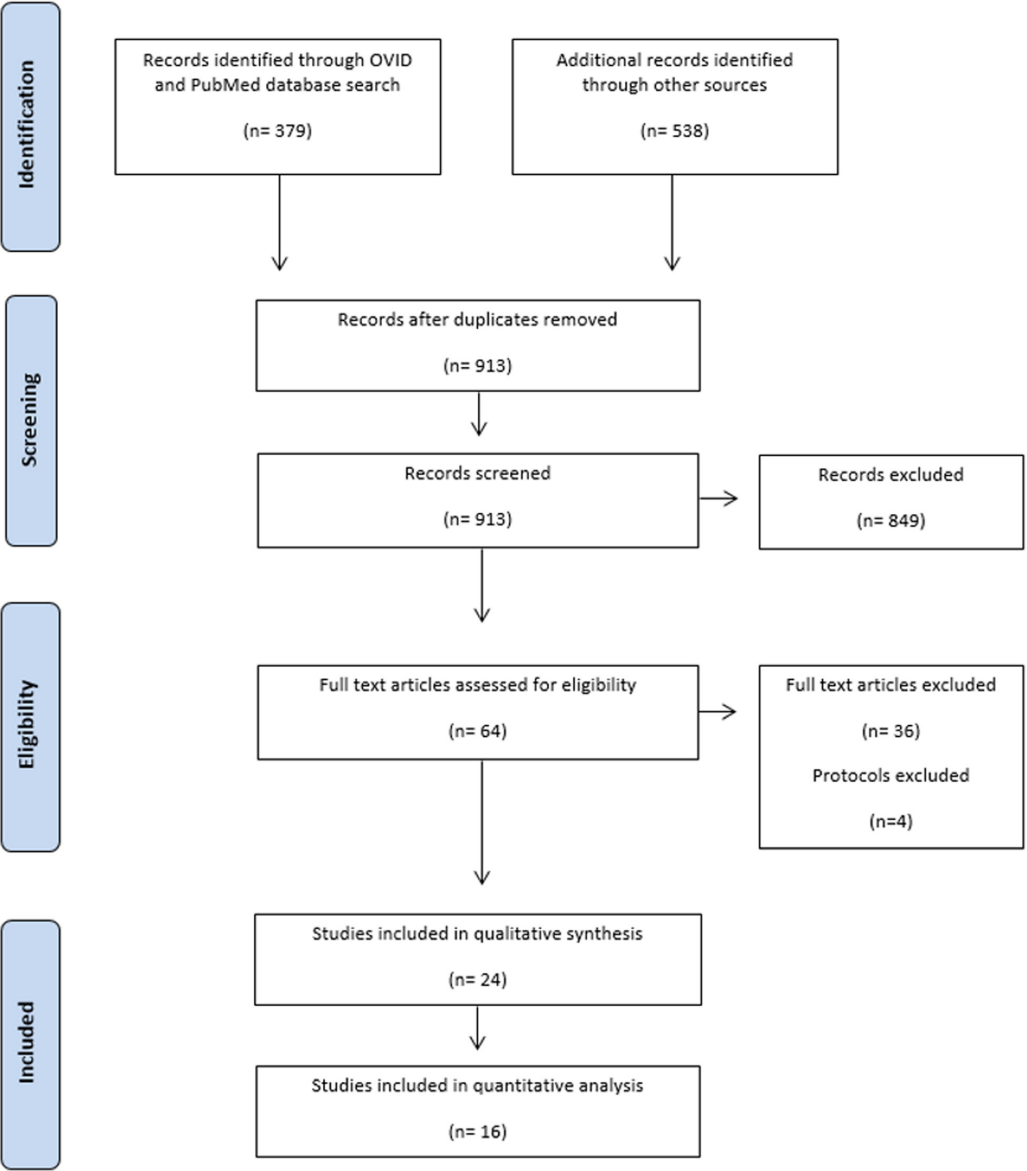
PRISMA flowchart of included studies

Sixty-four were considered potentially relevant to the review. Following retrieval of the full-text articles, 36 studies were excluded. Overall, 24 studies met the inclusion criteria (Fig. 1). Reasons for exclusion at the final stage are presented (Appendix 2). The study design, characteristics of participants, comparison groups, follow-up period, and the outcomes of the included studies are presented in Table 1.

**Table 1:** Characteristics of included trials (*n* = 24)

Study	Design	Participants (overall)	Intervention/comparison	Wear (part-time/full-time)	Follow-up period (mean ± SD)	Dental arch	Outcomes
Al-Nimri et al. 2009 [25]	Prospective cohort study	*n* = 62 (18 M, 44 F)	- 0.036″ round stainless steel fixed retainer (canines only) (*n* = 31; mean age, 20.23 ± 3.8 years) - 0.015″ multistrand fixed retainer (*n* = 31; mean age, 19.97 ± 4.2 years)		21.31 months 19.35 months	Mandibular anterior teeth	*Plaque Index*, *Gingival Index*, *retainer failure*, Oral Hygiene Index, Irregularity Index
Bazargani et al. 2012 [14]	RCT	*n* = 51 Overall mean age, 18.3 ± 1.3 years	- 0.0195″ multistrand fixed retainer with two-step bonded resin adhesive (*n* = 25) - 0.0195″ multistrand fixed retainer with non-resin adhesive (*n* = 26)		24.4 ± 4.7 months	Mandibular anterior teeth	*Retainer failure*, *calculus accumulation*, discoloration around composite pads
Störmann and Ehmer 2002 [15]	RCT	*n* = 98 Overall age range, 13–17 years	- 0.0195″ Respond® fixed retainer (*n* = 30) - 0.0215″ Respond® fixed retainer (*n* = 36) - Prefabricated fixed retainer (canines only) (*n* = 32)		24 months	Mandibular anterior teeth	*Bleeding on probing*, *Plaque Index*, *failure rate*, *aesthetic problems*, *patient discomfort*, Little’s irregularity index, occlusal discrepancies, intercanine width
Tynelius et al. 2014 [13]	RCT	*n* = 75 (30 M, 45 F) Overall mean age, 14.3 ± 1.5 years	- Vacuum-formed retainer in the maxilla and 0.7-mm spring hard wire fixed retainer in the mandibule (canines only) (*n* = 25)	Full-time for 2 days followed by part-time for 1 year. Every other night in the second year	24 months	Maxillary and mandibular dentition	*Cost-effectiveness and societal costs*
			- Vacuum-formed retainer in the maxilla and interproximal enamel reduction in the mandibular anterior teeth (*n* = 25)	Full-time for 2 days followed by part-time for 1 year. Every other night in the second year			
			- Prefabricated positioner (*n* = 25)	Part-time for 1 year, followed by every other night in the second year			
Torkan et al. 2014 [16]	RCT	*n* = 30 (10 M, 20 F)	- Fiber-reinforced resin composite fixed retainer (*n* = 15; mean age, 16.2 ± 1.9) - 0.0175″ Multistrand stainless steel fixed retainer (*n* = 15; mean age, 15.7 ± 2.1 years)		6 months	Maxillary and mandibular anterior teeth	*Plaque Index*, *Calculus Index*, *Gingival Index*, *bleeding on probing*, width of periodontal ligament
Sfondrini et al. 2014 [17]	RCT	*n* = 87 (35 M, 52 F) Overall average age, 24 years (14–62 years)	- 0.5-mm silanized-treated glass fiber-reinforced composite resin fixed retainer (*n* = 40) - 0.0175″ multistrand stainless steel fixed retainer (*n* = 47)		12 months	Mandibular anterior teeth	*Bond adhesive failure*
Ardeshna et al. 2011 [26]	Prospective cohort study	*n* = 56 (76 fixed retainers)	- 0.53- or 1.02-mm fiber-reinforced thermoplastic fixed retainer with polyethylene terephthalate glycol matrix resin		24 months	Maxillary anterior teeth (2 retainers), mandibular anterior teeth (21 retainers, 6 of them were bonded to canines only)	*Survival and failure rates*
			- 0.53- or 1.02-mm fiber-reinforced thermoplastic fixed retainer with polycarbonate matrix resin			Maxillary anterior teeth (14 retainers), mandibular anterior teeth (39 retainers, 5 of them were canines only)	
Salehi et al. 2013 [18]	RCT	*n* = 142 (59 M, 83 F) Overall age range, 14–28 years	- Polyethylene woven ribbon fixed retainer (*n* = 68; mean age, 18.1 ± 5.23 years) - 0.0175″ multistrand stainless steel fixed retainer (*n* = 74; mean age, 18.2 ± 4.81 years)		18 months	Maxillary and mandibular anterior teeth	*Survival and failure rates*
Hichens et al. 2007 [12]	RCT	*n* = 355 (350 questionnaires completed at 6 months) (155 M, 242 F)^a^ Overall mean age = 14–15 years	- Hawley retainer (*n* = 172)	Full-time for 3 months followed by part-time for 3 months	6 months	Maxillary and mandibular dentition	*Cost-effectiveness*, *patient satisfaction*, *failure rate*, Little’s irregularity index
			- Vacuum-formed retainer (*n* = 183)	Full-time for 1 week, followed by part-time			
Bolla et al. 2012 [39]	RCT	*n* = 85 (29 M, 56 F)	- Glass fiber-reinforced fixed retainer (*n* = 40; mean age for M, 23.4 years; mean age for F, 20.2 years) - 0.0175″ multistrand stainless steel fixed retainer (*n* = 45; mean age for M, 24.1 years; mean age for F, 22.6 years)		6 years	Maxillary 2-2 (14 retainers) and mandibular (34 retainers) anterior teeth Maxillary 2-2 (18 retainers) and mandibular (32 retainers) anterior teeth	*Bond failure and breakage of retainers*
Tacken et al. 2010 [31]	RCT	*n* = 274 (135 M, 139 F)^a^ Overall mean age, 14 years	- Glass fiber-reinforced fixed retainer (500 unidirectional glass fibers) (*n* = 45; mean age, 14.8 years ± 1.3 years) - Glass fiber-reinforced fixed retainer (1000 unidirectional glass fibers) (*n* = 48; mean age, 14.6 years ± 2.7 years) - 0.0215″ coaxial fixed retainer (*n* = 91; mean age, 15 years ± 1.3 years) - Untreated control (*n* = 90)		24 months	Maxillary 2-2 and mandibular anterior teeth	*Failure rate*, *modified gingival index (MGI)*, *bleeding on probing*, *Plaque Index (PI)*
Bovali et al. 2014 [19]	RCT	*n* = 63 (28 M, 35 F) Overall age range: 12–38 years	- Direct bonding of 0.0215″ multistrand stainless steel fixed retainer (*n* = 31; mean age, 19.8 ± 6.5 years) - Indirect bonding of 0.0215″ multistrand stainless steel fixed retainer (*n* = 32; mean age, 17.2 ± 3.1 years)		6 months	Mandibular anterior teeth	*Failure rate*, time to fit retainers
Pandis et al. 2013 [20]	RCT	*n* = 220 (60 M, 160 F) Overall median age, 16 (IQR 2) years Overall age range, 12–47 years	- 0.022″ multistrand stainless steel fixed retainer bonded with chemical-cured composite (*n* = 110; median age, 16 (IQR 2) years) - 0.022″ multistrand stainless steel fixed retainer bonded with light-cured composite (*n* = 110; median age, 16 (IQR 2) years)		Median follow-up period: 2.19 years Range, 0.003–3.64 years	Mandibular anterior teeth	*Failure rate*, adhesive remnant index scores
Sun et al. 2011 [11]	RCT	*n* = 111 Overall mean age, 14.7 years Overall age range, 12–17 years	- Hawley retainer (*n* = 56)	Full-time	12 months	Maxillary and mandibular dentition	*Survival and failure rates*
			- Vacuum-formed retainer (*n* = 55)	Full-time			
Xu et al. 2011 [40]	RCT	*n* = 40 (16 M, 29 F) Overall mean age, 13–16 years	- Vacuum-formed retainer (*n* = 25)	Full-time	12 months	Maxillary and mandibular dentition	Overjet, overbite, intercanine width, intermolar width, Little’s irregularity index, *Calculus Index scores*, *failure rate*
			- 0.0195″ multistrand stainless steel fixed retainer with Hawley retainer (*n* = 15)	Part-time			
Rose et al. 2002 [41]	RCT	*n* = 20 (12 M, 8 F) Overall mean age, 22.4 ± 9.7 years	- 1-mm polyethylene woven ribbon fixed retainer (*n* = 10) - 0.0175″ multistrand stainless steel fixed retainer (*n* = 10)		24 months	Mandibular anterior teeth	*Patient acceptance and preference*, *survival of retainers*, *amount of calculus*, demineralisation, caries
Liu et al. 2010 [23]	RCT	*n* = 60	- 0.75-mm fiber-reinforced composite fixed retainer (*n* = 30) - 0.9-mm multistrand stainless steel fixed retainer (*n* = 30)		12 months	Mandibular anterior teeth	*Bleeding index*, *pocket depth*, *failure rate*
Taner et al. 2012 [27]	Prospective cohort study	*n* = 66 (14 M, 52 F)	- Direct bonding of 0.016″ × 0.022″ multistrand stainless steel dead soft wire fixed retainer (*n* = 32; mean age, 15.96 ± 3.21 years) - Indirect bonding of 0.016″ × 0.022″ multistrand stainless steel dead soft wire fixed retainer (*n* = 34; mean age, 19.44 ± 6.79 years)		6 months	Mandibular anterior teeth	*Failure rate*
Artun et al. 1997 [28]	Prospective cohort study	*n* = 49	- 0.032″ plain fixed retainer (canines only) (*n* = 11)		3 years	Mandibular anterior teeth	Little’s irregularity index, *failure rate*, *Plaque Index*, *Calculus Index*, *Gingival Index*, *probing attachment level*
			- 0.032″ spiral wire fixed retainer (canines only) (*n* = 13)				
			- 0.0205″ spiral wire fixed retainer (*n* = 11)				
			- Removable retainer (*n* = 14)	Unclear			
Scribante et al. 2011 [24]	RCT	*n* = 34 (9 M, 25 F) Overall mean age, 14.3 years	- 0.0175″ multistrand stainless steel fixed retainer - Polyethylene fiber-reinforced resin composite fixed retainer		12 months	Mandibular anterior teeth	*Failure rate*, *patient satisfaction of the aesthetic result*
Zachrisson, 1977 [29]	Prospective cohort study	*n* = 43 (14–17 years)	- 0.032″ or 0.036″ blue Elgiloy fixed retainer bonded using a holding wire (canines only) (*n* = 22)		Mean, 15.7 months; Range, 12–30 months	Mandibular anterior teeth	*Failure rate*, calculus accumulation
			- 0.032″ or 0.036″ blue Elgiloy fixed retainer bonded using a steel ligature (canines only) (*n* = 21)				
Heier et al. 1997 [30]	Prospective cohort study	*n* = 36 Overall mean age, 16.3 years Overall age range, 12.8–21.1 years	- 0.0175″ multistrand stainless steel fixed retainer (*n* = 22)		6 months	Maxillary and mandibular anterior teeth Maxillary and mandibular dentition	*Modified gingival index*, *bleeding on probing*, *Plaque Index*, *Calculus Index*, gingival crevicular fluid flow
			- Hawley retainer (*n* = 14)	Unclear			
Sobouti et al. 2016 [21]	RCT	*n* = 128 (60 M, 68 F) Overall mean age, 18 ± 3.6 years Overall age range, 13–25 years	- Fiber-reinforced composite fixed retainer (*n* = 42; mean age, 18.5 ± 3.6 years) - 0.0175″ flexible spiral wire fixed retainer (*n* = 41; mean age, 18.4 ± 3.7 years) - 0.0009″ dead soft twisted wires fixed retainer (*n* = 45; mean age, 17 ± 3.3 years)		24 months	Mandibular anterior teeth	*Survival and failure rates*
O’Rouke et al. 2016 [22]	RCT	*n* = 82 (23 M, 59 F)	- Vacuum-formed retainer (*n* = 40, mean age: 16.95 ± 2.02 years) - 0.0175″ stainless steel coaxial fixed retainer (*n* = 42, Mean age: 18.47 ± 4.41 years)	Full-time for 6 months, followed by part-time for 6 months, then for every other night in the second year	18 months	Mandibular dentition	Little’s irregularity index, intercanine width, intermolar width, arch length**,** *failure rate*

^a^Overall sample

#### Risk of bias/methodological quality of included studies

**Fig. 2:**
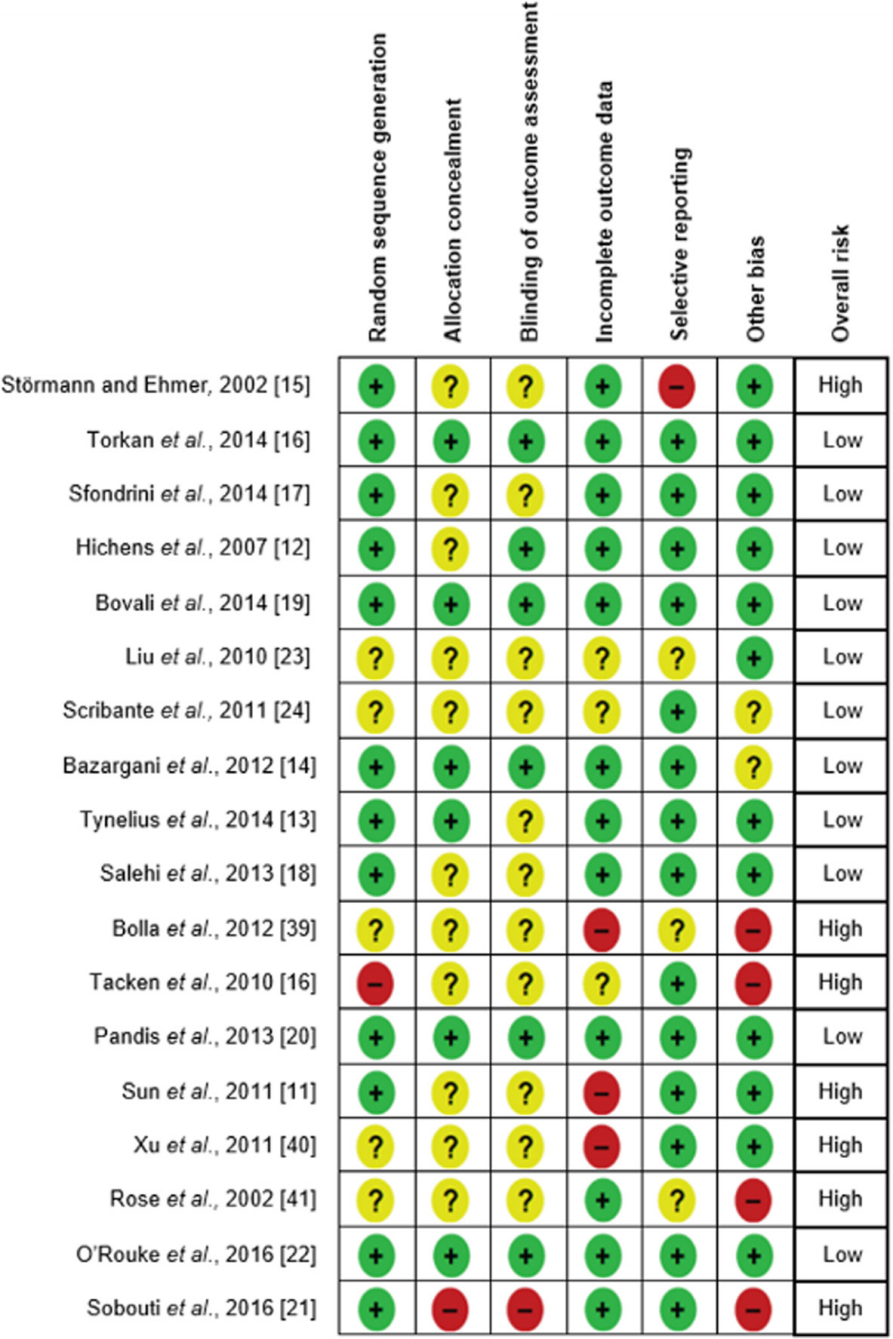
Risk of bias for included randomized controlled trials. Low risk of bias (*green*). Unclear risk of bias (*yellow*). High risk of bias (*red*)

**Fig. 3:**
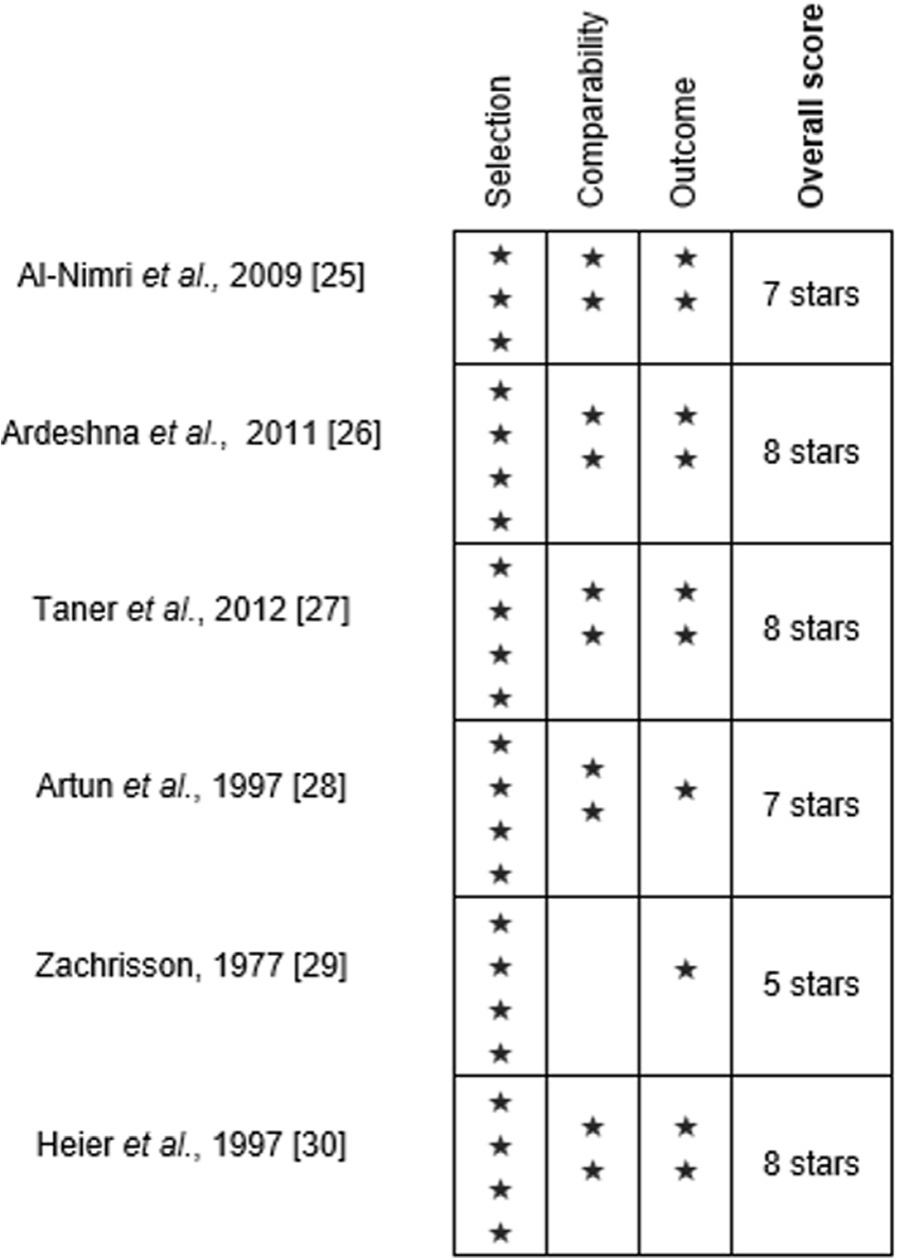
Newcastle-Ottawa Scale scores for non-randomized studies

The random sequence generation was adequately performed in 12 studies [11–22]. The assessor was adequately blinded in six trials [13, 14, 16, 19, 20, 22]. Overall, 11 randomized clinical trials were judged to be of low risk of bias (Fig. 2) [12–14, 16–20, 22–24]. All six prospective cohort studies [25–30] (Fig. 3) were deemed to be of high quality in terms of sample selection, except for one study [25] which did not demonstrate the absence of pre-existing periodontal disease. Assessment of the outcome was deemed satisfactory in all but two studies [28, 29]. Overall, five prospective cohort studies were judged to be of moderate to high quality [25–28, 30].

#### Periodontal outcomes

Of the included trials, only seven trials assessed periodontal outcomes (Tables 2 and 3) [14, 16, 23, 25, 28, 30, 31]. Four of these were randomized controlled trials [14, 16, 23, 31], and the other three were prospective cohort studies [25, 28, 30]. Two trials did not report baseline scores [14, 25], and another two studies reported the periodontal outcome with no distinction made between maxillary and mandibular measurements [30, 31].

**Table 2:** Periodontal outcomes

	Intervention	Periodontal outcomes	Index	Arch	Teeth	Tooth surfaces
Al-Nimri et al. 2009 [25]	- 0.036″ round stainless steel fixed retainer (canines only) - 0.015″ multistrand fixed retainer	Plaque Index	0 absence 1 on probe 2 visible 3 abundant	Mandible	3-3	Labial/lingual/mesial/distal
		Gingival Index	0 absence 1 mild 2 moderate 3 severe	Mandible	3-3	Labial and lingual
		Calculus	Part of Oral Hygiene Index Tooth with the highest score determine the index score for the segment (6 segments)	Maxilla and mandible	All teeth except mandibular labial segment	Labial and lingual
Bazargani et al. 2012 [14]	- 0.0195″ multistrand fixed retainer with two-step bonded resin adhesive - 0.0195″ multistrand fixed retainer with non-resin adhesive	Calculus	Present/absent	Mandible	3-3	Lingual
Torkan et al. 2014 [16]	- Fiber-reinforced resin composite fixed retainer - 0.0175″ multistrand stainless steel fixed retainer	Plaque Index	Using disclosing 0 absence 1 visible on the probe 2 visible 3 abundant	Maxilla and mandible	3-3	Lingual
		Calculus Index	0 absence 1 up to 1/3 2 up to 2/3 3 > 2/3	Maxilla and mandible	All teeth	Unclear
		Gingival Index	0 absence 1 mild 2 moderate 3 severe	Maxilla and mandible	Unclear	Lingual
		Bleeding on probing	Present/absent	Maxilla and mandible	3-3	Unclear
Tacken et al. 2010 [31]	- Glass fiber-reinforced fixed retainer (500 unidirectional glass fibers) - Glass fiber-reinforced fixed retainer (1000 unidirectional glass fibers) - 0.0215″ coaxial fixed retainer - Untreated control	Gingival Index	0 absence 1 mild (localized) 2 mild (generalized) 3 moderate 4 severe	Unclear	Unclear	Unclear, 3 sites/tooth: mesial, distal, central
		Bleeding on probing	0 no bleeding 1 point bleeding 2 abundant bleeding	Unclear	Unclear	Unclear, 3 sites/tooth: mesial, distal, central
		Plaque Index	Using disclosing 0 no plaque 1 spots at the cervical margin 2 thin band at the cervical margin 3 gingival 1/3 4 gingival 2/3 5 > gingival 2/3	Unclear	Unclear	Unclear, 3 sites/tooth: mesial, distal, central
Artun et al. 1997 [28]	- 0.032″ plain fixed retainer (canines only) - 0.032″ spiral wire fixed retainer (canines only) - 0.0205″ spiral wire fixed retainer - Removable retainer	Plaque Index	0 absence 1 on probe 2 visible 3 abundant	Mandible	3-3	Lingual, mesial, distal
		Gingival Index	0 absence 1 mild 2 moderate 3 severe	Mandible	3-3	Lingual, mesial, distal
		Calculus Index	0 absence 1 supragingival calculus not more than 1 mm 2 gingival 1/3 3 > gingival 2/3	Mandible	3-3	Lingual, mesial, distal
		Pocket depth	Mean attachment loss	Mandible	3-3	Lingual
Liu et al. 2010 [23]	- 0.75-mm fiber-reinforced composite fixed retainer - 0.9-mm multistrand stainless steel fixed retainer	Pocket depth	Scores added together	Mandible	3-3	Lingual (3 sites/tooth)
		Bleeding on probing	Scores added together	Mandible	3-3	Lingual (3 sites/tooth)
Heier et al. 2010 [30]	- 0.0175″ multistrand stainless steel fixed retainer - Hawley retainer	Gingival Index	0 absence 1 mild (localized) 2 mild (generalized) 3 moderate 4 severe	Maxilla and mandible	3-3	Labial, lingual, interdental labial, interdental lingual
		Bleeding on probing	0 absence 1 point bleeding 2 profuse	Maxilla and mandible	3-3	Labial, lingual, interdental labial, interdental lingual
		Plaque Index	Using disclosing 0 no plaque 1 spots at the cervical margin 2 thin band at the cervical margin 3 gingival 1/3 4 gingival 2/3 5 > gingival 2/3	Maxilla and mandible	3-3	Labial, lingual
		Calculus Index	Overall mean score	Maxilla and mandible	3-3	Labial, lingual (3 sites/surface)

**Table 3:** Periodontal outcomes including the follow-up periods

Study	Intervention	Plaque Index				Gingival Index				Calculus	Bleeding on probing				Probing attachment level
Al-Nimri et al. 2009 [25]	- 0.036″ Round stainless steel fixed retainer (canines only) (*n* = 31)	Mean after at least 12 months, 1.02 ± 0.52				Mean after at least 12 months, 1.19 ± 0.44									
	- 0.015″ multistrand fixed retainer (*n* = 31)	Mean after at least 12 months, 1.21 ± 0.48				Mean after at least 12 months, 1.34 ± 0.39									
Bazargani et al. 2012 [14]	- 0.0195″ multistrand fixed retainer with two-step bonded resin adhesive (*n* = 25)									4 % (2 years)					
	- 0.0195″ multistrand fixed retainer with non-resin adhesive (*n* = 26)									31 % (2 years)					
Torkan et al. 2014 [16]	- Fiber-reinforced composite resin fixed retainer (*n* = 15)	Maxilla: median 0 (baseline), 1.66 (6 months) Mandible: median 0.91 (baseline), 2 (6 months)				Maxilla: median 0.5 (baseline), 1 (6 months) Mandible: median 0.33 (baseline) 1 (6 months)				Maxilla: Median 0 (baseline and 6 months) Mandible: Median 0 (baseline), 0.33 (6 months)	Maxilla: Median 0.16 (baseline), 0.5 (6 months) Mandible: Median 0 (baseline), 0.66 (6 months)				
	- 0.0175″ multistrand stainless steel fixed retainer (*n* = 15)	Maxilla: median 0.33 (baseline), 0.66 (6 months) Mandible: median 0.33 (baseline), 0.91 (6 months)				Maxilla: median 0 (baseline), 0.83 (6 months) Mandible: median 0.16 (baseline), 0.41 (6 months)				Maxilla and mandible: Median 0 (baseline and 6 months)	Maxilla: median 0 (baseline), 0.5 (6 months) Mandible: median 0 (baseline) 0.33 (6 months)				
Tacken et al. 2010 [31]	- Glass fiber-reinforced fixed retainer (500 unidirectional glass fibers) (*n* = 45)	6 months, 1.88 ± 0.74	12 months, 2.32 ± 0.93	18 months, 2.25 ± 0.78	24 months, 2.11 ± 0.73	6 months, 1.20 ± 0.43	12 months, 1.00 ± 0.30	18 months, 1.28 ± 0.36	24 months, 1.51 ± 0.45		6 months, 0.72 ± 0.22	12 months, 0.89 ± 0.19	18 month, 0.82 ± 0.23	24 months, 1.00 ± 0.35	
	- Glass fiber-reinforced fixed retainer (1000 unidirectional glass fibers) (*n* = 48)	6 months, 2.03 ± 0.84	12 months, 2.12 ± 0.77	18 months, 2.48 ± 0.69	24 months, 2.18 ± 0.79	6 months, 1.09 ± 0.46	12 months, 1.09 ± 0.34	18 months, 1.20 ± 0.33	24 months, 1.55 ± 0.37		6 months, 0.76 ± 0.18	12 months, 0.81 ± 0.21	18 months, 0.89 ± 0.23	24 months, 1.06 ± 0.29	
	- 0.0215″ coaxial fixed retainer (*n* = 91)	6 months, 1.74 ± 0.92	12 months, 2.09 ± 0.82	18 months, 2.07 ± 0.76	24 months, 2.14 ± 0.78	6 months, .0.71 ± 0.29	12 months, 0.61 ± 0.29	18 months, 0.70 ± 0.27	24 months, 0.98 ± 0.54		6 months, 0.46 ± 0.18	12 months, 0.55 ± 0.19	18 months, 0.57 ± 0.21	24 months, 0.84 ± 0.38	
Liu et al. 2010 [23]	- 0.75-mm fiber-reinforced composite fixed retainer (*n* = 30)										Baseline, 3.50 6 months, 10.17 12 months, 11.12				Baseline, 6.33 6 months: 8.51 mm 12 months: 9.24 mm
	- 0.9-mm multistrand stainless steel fixed retainer (*n* = 30)										Baseline, 3.67; 6 months, 8.89; 12 months, 9.24				Baseline, 5.92 6 months: 8.08 mm 12 months: 8.92 mm
Artun et al. 1997 [28]	- 0.032″ plain fixed retainer (canines only) (*n* = 11)	Baseline, 0.32 3 years, 0.06				Baseline, 1.01 3 years, 0.66				Baseline, 16.67 3 years, 3.33					Mean attachment loss at 3 years, 0.85 mm
	- 0.032″ spiral fixed retainer (canines only) (*n* = 13)	Baseline, 0.17 3 years, 0.10				Baseline, 0.95 3 years, 0.49				Baseline: 8.64 3 years, 3.09					Mean attachment loss at 3 years, 0.63 mm
	- 0.0205″ spiral wire fixed retainer (*n* = 11)	Baseline, 0.26 3 years, 0.13				Baseline, 1.14 3 years, 0.39				Baseline, 17.36 3 years, 17.36					Mean attachment loss at 3 years, 0.62 mm
	- Removable retainer (*n* = 14)	Baseline, 0.31 3 years, 0.13				Baseline, 1.08 3 years, 0.77				Baseline, 9.52 3 years, 8.33					Mean attachment loss at 3 years, 0.72 mm
Heier et al. 2010 [30]	- 0.0175″ multistrand stainless steel fixed retainer (*n* = 22)	Baseline, 2.78 6 months, 3.03				Baseline, 0.79 6 months, 0.40				Baseline and 6 months, 0.20	Baseline, 0.32 6 months, 0.23				
	- Hawley retainer (*n* = 14)	Baseline, 2.78 6 months, 2.52				Baseline, 0.80 6 months, 0.74				Baseline, 0.05 6 months, 0.06	Baseline, 0.34 6 months, 0.22				

No significant difference was found between mandibular stainless steel fixed retainers bonded to the anterior teeth and canines only in terms of periodontal outcomes, at 12-month and 3-year follow-ups in two studies [25, 28]. With regard to periodontal outcomes of mandibular Hawley retainers in comparison to mandibular stainless steel fixed retainers, no significant difference was found at 3-year follow-up [28]. When mandibular fiber-reinforced composite was compared to mandibular stainless steel fixed retainers, no significant difference in probing depths, bleeding on probing, and calculus scores at 6-month follow-up was found [16, 23]. Probing depths and bleeding on probing were further measured at 12-month follow-up and showed no significant difference between the two groups [23]. However, gingival and plaque indices scores were found to be higher in maxillary and mandibular fiber-reinforced composite compared to stainless steel fixed retainers at 6-month follow-up [16]. Very few overlapping studies were identified, however. Meta-analysis was therefore not possible in view of heterogeneity.

In terms of the natural history of periodontal changes related to stainless steel fixed retainers, plaque and gingival indices scores on the lingual surfaces of mandibular anterior teeth increased from baseline to 6 months follow-up; however, this was not statistically significant [16]. At 3-year follow-up, plaque and gingival indices scores remained low [28]. No significant changes in Calculus Index scores at 6-month [16] and 3-year follow-ups [28] were observed in two studies. Bleeding on probing scores for stainless steel fixed retainer increased at both 6 months [16, 23] and 12 months [23] from baseline, although only one study found this to be statistically significant [23]. Similar patterns were observed for fixed fiber-reinforced composite retainers [16, 23]. Conversely, plaque, calculus, and gingival indices scores reduced at 3-year follow-up in relation to the lingual of the mandibular anterior teeth with Hawley retainers [28]. However, Gingival Index scores were shown to increase on the buccal surfaces of maxillary and mandibular anterior teeth in one study at 6-month follow-up [30].

#### Survival and failure rates of retainers

The survival rate of fixed retainers was reported over 12 to 24 months [18, 24, 26]. In terms of retainer material, one study found fiber-reinforced thermoplastic fixed retainer with polyethylene terephthalate glycol matrix resin survived significantly less than fiber-reinforced thermoplastic fixed retainer with polycarbonate matrix resin [26]. Two other studies found no significant difference in the survival rate of multistrand stainless steel fixed and esthetic retainers made of polyethylene woven ribbon or polyethelene fiber-reinforced resin composite [18, 24]. No statistical difference was found in the survival rate between maxillary and mandibular fixed retainers [18, 26]. Interestingly, in one study, the survival rate of fiber-reinforced thermoplastic fixed retainers was directly related to the thickness of the wire and the number of teeth bonded [26].

**Fig. 4:**
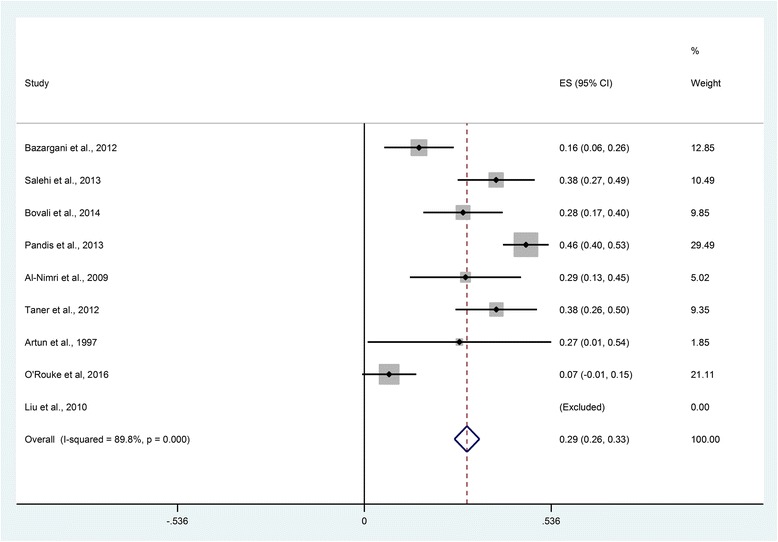
Risk of failure of mandibular stainless steel fixed retainers bonded from canine to canine

**Fig. 5:**
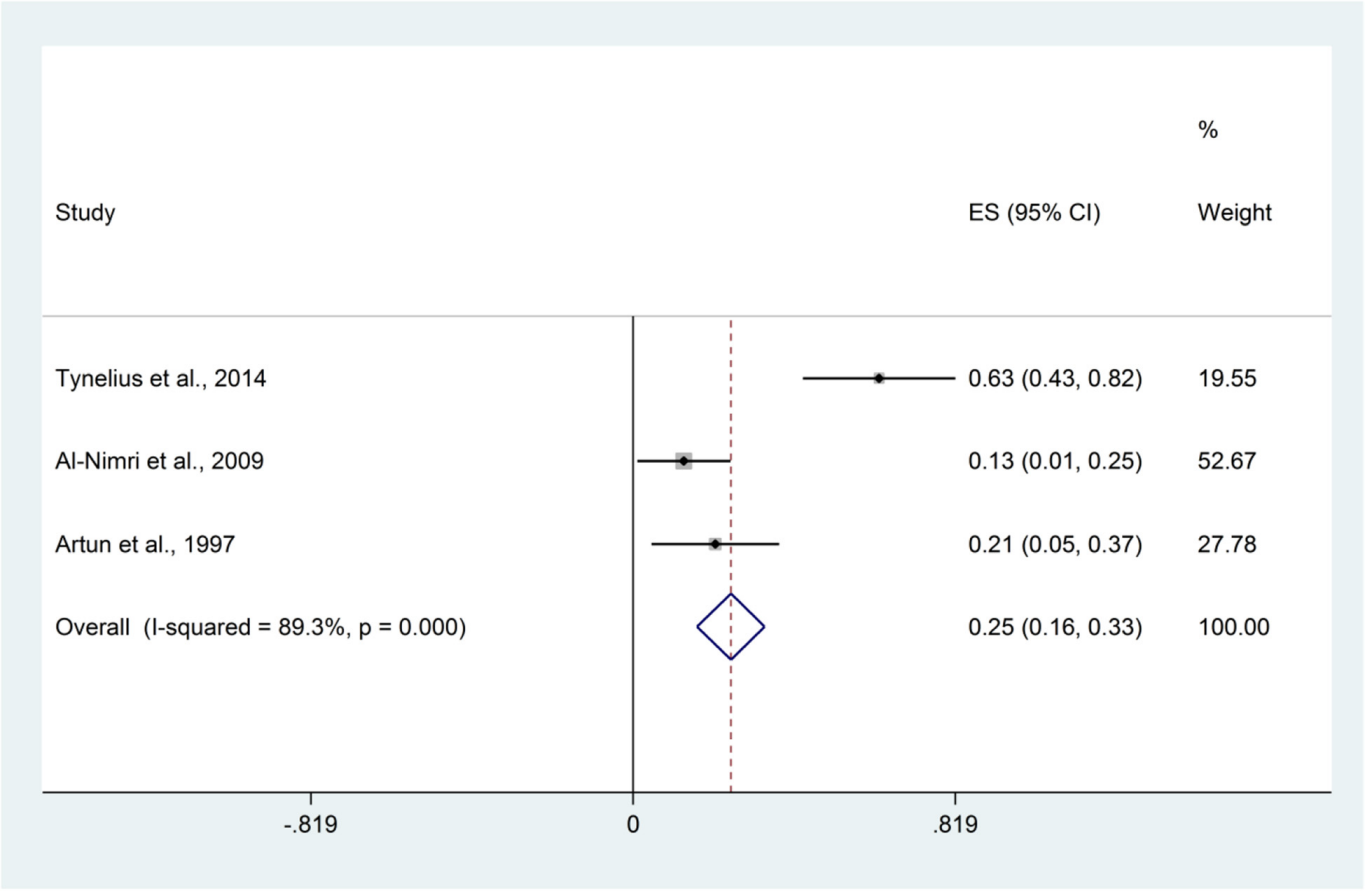
Risk of failure of mandibular stainless steel fixed retainers bonded to canines only

All the studies that involved mandibular stainless steel retainers reported failures per patient [13, 14, 17–20, 22–25, 27, 28], except for two studies in which the failure was reported per tooth [17, 24] (Table 4). The mean failure risk for mandibular stainless steel fixed retainers bonded to canine to canine was 0.29 (95 % confidence interval [CI], 0.26, 0.33) based on nine studies (*n* = 555) (Fig. 4). The follow-up period ranged from 6 to 36 months. Similarly, the failure risk for mandibular stainless steel fixed retainers bonded to canines was 0.25 (95 % CI, 0.16, 0.33) based on three studies [13, 25, 28] (*n* = 79) over a follow-up period of 12 to 36 months (Fig. 5). Considerable statistical heterogeneity was noted in both analyses (*I*-squared = 89 %) reflecting high levels of inconsistency and limited numbers of events. A meta-regression shows that follow-up period was not a predictor of failure rate for mandibular stainless steel fixed retainers (*P* = 0.938).

**Table 4:** Survival and failure rates of fixed and removable retainers

Study	Intervention	Survival rate	Failure rate
Al-Nimri et al. 2009 [25]	- 0.036″ round stainless steel fixed retainer (canines only)		4/31 (13 %)
	- 0.015″ multistrand fixed retainer		9/31 (29 %)
Bazargani et al. 2012 [14]	- 0.0195″ multistrand fixed retainer with two-step bonded resin adhesive		1/25 (4 %)
	- 0.0195″ multistrand fixed retainer with non-resin adhesive		7/26 (27 %)
Tynelius et al. 2014 [13]	- Vacuum-formed retainer in the maxilla and 0.7-mm spring hard wire fixed retainer in the mandible (canines only)		2/24 (8.3 %) vacuum-formed retainer, 15/24 (62.5 %) fixed retainer
	- Vacuum-formed retainer in the maxilla and interproximal enamel reduction in the mandibular anterior teeth		3/25 (12 %)
	- Prefabricated positioner		0/25 (0 %)
Sfondrini et al. 2014 [17]	- 0.5-mm silanized-treated glass fiber-reinforced composite resin fixed retainer		27/240 bonded teeth (11.25 %)
	- 0.0175″ multistrand stainless steel fixed retainer		50/282 bonded teeth (17.73 %)
Ardeshna et al. 2011 [26]	- 0.53- or 1.02-mm fiber-reinforced thermoplastic fixed retainer with polyethylene terephthalate glycol matrix resin	Median, 2.97 months	22/23 (95.6 %)
	- 0.53- or 1.02-mm fiber-reinforced thermoplastic fixed retainer with polycarbonate matrix resin	Median, 11.37 months	32/53 (60.3 %)
Salehi et al. 2013 [18]	- Polyethylene woven ribbon fixed retainer	Maxilla: mean 13.96 ± 4.53 months Mandible: mean 14.26 ± 4.70 months	34/68 in the maxilla (50 %), 29/68 in the mandible (42.6 %)
	- 0.0175″ multistrand stainless steel fixed retainer	Maxilla: mean 15.34 ± 4.04 months Mandible: mean 15.61 ± 3.61 months	27/74 in the maxilla (36.5 %), 28/74 in the mandible (37.8 %)
Hichens et al. 2007 [12]	- Hawley retainer		40/344 (11.6 %)
	- Vacuum-formed retainer		20/366 (17 %)
Bovali et al*.* 2014 [19]	- Direct bonding of 0.0215″ multistrand stainless steel fixed retainer		7/29 (24.1 %)
	- Indirect bonding of 0.0215″ multistrand stainless steel fixed retainer		10/31 (32.2 %)
Pandis et al. 2013 [20]	- 0.022″ multistrand stainless steel fixed retainer bonded with chemical-cured composite		47/110 (42.7 %)
	- 0.022″ multistrand stainless steel fixed retainer bonded with light-cured composite		55/110 (50 %)
Liu et al. 2010 [23]	- 0.75-mm fiber-reinforced composite fixed retainer		0/30 (0 %)
	- 0.9-mm multistrand stainless steel fixed retainer		0/30 (0 %)
Taner et al. 2012 [27]	- Direct bonding 0.016″ × 0.022″ multistrand stainless steel dead soft wire fixed retainer		15/32 (46.8 %)
	- Indirect bonding 0.016″ × 0.022″ multistrand stainless steel dead soft wire fixed retainer		10/34 (29.4 %)
Artun et al. 1997 [28]	- 0.032″ plain fixed retainer (canines only)		1/11 (9.1 %)
	- 0.032″ spiral fixed retainer (canines only)		4/13 (30.7 %)
	- 0.0205″ spiral wire fixed retainer		3/11 (27.27 %)
	- Removable retainer		2/14 (14.28 %)
Scribante et al. 2011 [24]	- 0.0175″ multistrand stainless steel fixed retainer		23/102 bonded teeth (23 %)
	- Polyethylene fiber-reinforced resin composite fixed retainer		13/90 bonded teeth (14 %)
O’Rouke et al. 2016 [22]	- Vacuum-formed retainer		
	- 0.0175″ stainless steel coaxial fixed retainer		3/42 (7.14 %)

One study reporting failure rates of mandibular Hawley retainers was unclear regarding the stipulated duration of wear [28]. However, two studies found around 12 % failure over a period of 6 months and 14 % at 3-year follow-up [12, 28]. Similarly, the failure rate for maxillary vacuum-formed retainers was found to be 10 % over 2 years [13], while a further study reported a higher rate of 17 % over 6 months [12].

#### Patient-reported outcomes and cost-effectiveness

Patient-reported outcomes were reported in two studies [12, 24] (Table 5). Removable retainers were found to be associated with discomfort, with those in the Hawley retainer group reporting higher levels of embarrassment in terms of speech and esthetics [12].

**Table 5:** Patient-reported outcomes and cost-effectiveness

Study	Intervention	Patient-reported outcomes	Cost-effectiveness	
Tynelius et al. 2014 [13]	- Vacuum-formed retainer in the maxilla and 0.7-mm spring hard wire fixed retainer in the mandible (canines only)		Costs of scheduled appointments, €12,425	Costs of unscheduled appointments, €804
	- Vacuum-formed retainer in the maxilla and interproximal enamel reduction in the mandibular anterior teeth		Costs of scheduled appointments, €11,275	Costs of unscheduled appointments, €303
	- Prefabricated positioner		Costs of scheduled appointments, €10,500	Costs of unscheduled appointments, none
Hichens et al. 2007 [12]	- Hawley retainer	Embarrassment: 29/168 (17 %) Discomfort: 109/168 (65 %)	Mean cost to the NHS, €152 (€150.86, €153.15) per patient Mean cost to the orthodontic practice, −€1.00 (−€1.78, −€0.22) per patient Mean cost to the patient, €11.63 (€9.67, €13.59) per patient	
	- Vacuum-formed retainer	Embarrassment: 13/182 (7 %) Discomfort: 112/182 (62 %)	Mean cost to the NHS, €122.02 (€120.84, €123.21) per patient Mean cost to the orthodontic practice, −€34.00 (−€34.57, −€33.34) per patient Mean cost to the patient, €6.92 (€5.29, €8.53) per patient	
Scribante et al. 2011 [24]	- 0.0175″ multistrand stainless steel fixed retainer	Mean, 8.24 ± 1.39; median, 8.50; range, (4.50–10.0) (using visual analog scale)		
	- Polyethylene fiber-reinforced resin composite fixed retainer	Mean, 9.73 ± 0.42; median, 10.00; range, (9.00–10.0) (using visual analog scale)		

In terms of cost-effectiveness (Table 5), vacuum-formed retainers were found to be significantly more cost-effective than Hawley retainers within the National Health Service over a 6-month retention period [12]. One study, over 2 years, found interproximal reduction as a retention method and positioners to be more cost-effective than mandibular stainless steel fixed retainers bonded to canines [13].

### Discussion

This systematic review found a lack of evidence to endorse the use of one type of orthodontic retainer based on their effect on periodontal health, survival and failure rates, patient-reported outcomes, and cost-effectiveness. Largely, this finding can be attributed to a lack of high-quality, relevant research. In this respect, the results of the present systematic review are in line with previous systematic reviews [3, 9, 10]. Interestingly, it was found that failure of fixed stainless steel mandibular retainers was not directly related to the duration of follow-up. This suggests that other factors including the influence of operator technique and experience might override the effects of retainer design or materials, although follow-up did not extend beyond 3 years in the present review.

Generally, relatively minor changes in periodontal parameters were reported; however, given that most studies did not incorporate an untreated control, or indeed a control group without retention, it was unclear whether these changes were attributable to the intervention or temporal changes, in isolation. As such additional research including prospective cohort studies with matched controls incorporating baseline assessment would be helpful in providing more conclusive information. It is worthy of mention that the current standard of care is to recommend bonded retention to preserve orthodontic correction in those with a history of periodontal disease as these patients are known to be particularly susceptible to post-treatment changes [32, 33]. It is therefore important that there is greater clarity in relation to the compatibility of fixed retention with periodontal health and indeed on variations that may facilitate maintenance of optimal hygiene.

A minimum follow-up period of 6 months was set to distinguish between gingival inflammation associated with fixed orthodontic treatment and periodontal side-effects related to the orthodontic retainers [34]. Previous reviews have stipulated a minimum observation period of 3 months [3, 9] to 2 years [10]. However, a 3-month period might be insufficient to allow for the resolution of inflammatory changes related to the presence of active appliances. Using a minimum of 2-year observation period risks omission of a considerable amount of relevant research. Moreover, in this review, just one study focusing on periodontal outcomes involved follow-up in excess of 2 years [28]. It is therefore clear that the prolonged effect of orthodontic retention on periodontal health has not been adequately addressed in prospective research.

Intuitively, a significant difference in patient-reported outcomes and experiences could be expected with fixed or removable retainers in view of differences in appearance, size, and requirement for compliance. Notwithstanding this, only two studies reported on satisfaction with the appearance of retainers or on levels of associated embarrassment or discomfort [12, 24]. This tendency for researchers to concentrate on objective, often clinician-centered outcomes has recently been documented both within orthodontics and general dental research more broadly [35, 36]. Further studies incorporating patient-reported outcomes are therefore necessary to provide a more holistic assessment of benefits, harms, and experiences associated with orthodontic retainers.

While the primary focus of this review was to compare the effectiveness of retainer types, it was also possible to generate epidemiological data on the risk of failure of fixed retainers based on the primary studies. Failure risk of 0.29 was found for fixed wires bonded to the six anterior teeth and approximately one-quarter of retainers bonded to mandibular canines only, based on observation periods of 6 months to 3 years. This data highlights that the risk of failure is considerable and that fixed retention does not guarantee prolonged stability. Similar findings have been observed in observational studies [2]. The onus on realistic treatment planning with due consideration for placement of teeth into a zone of relative stability therefore remains [37].

Attempts were made to identify all trials meeting the inclusion criteria in the present review with no restrictions based on either publication date or language. Furthermore, we planned to include both prospective cohort studies and randomized controlled trials. Cohort studies were included, in particular, to permit assessment of periodontal outcomes as they are more likely to involve more prolonged periods of follow-up, which may be necessary to reveal the extent of prolonged periodontal effects. Meta-analysis was not undertaken in view of the clinical heterogeneity between the limited number of included studies, which made statistical pooling inappropriate in relation to periodontal health, survival and failure rates, patient-reported outcomes, and cost-effectiveness. This inability to undertake meta-analysis is common to many orthodontic systematic reviews with meta-analysis found in just 27 % of 157 reviews over a 14-year period with a median of just 4 studies for those that did incorporate meta-analysis [38]. The onus on producing high-quality primary research studies in orthodontics remains.

## Conclusions

There is a lack of high-quality evidence to endorse the use of one type of orthodontic retainer based on their effect on periodontal health, risk of failure, patient-reported outcomes, and cost-effectiveness. Further well-designed prospective studies are therefore required to provide further definitive information in relation to the benefits and potential harms of prolonged retention.

## Appendix 1

Database: Ovid MEDLINE(R) <1946 to Present>

Search Strategy:

1 RANDOMIZED CONTROLLED TRIAL.pt. (413632)

2 CONTROLLED CLINICAL TRIAL.pt. (91880)

3 RANDOM ALLOCATION.sh. (86446)

4 DOUBLE BLIND METHOD.sh. (135365)

5 SINGLE BLIND METHOD.sh. (21423)

6 or/1-5 (586980)

7 (ANIMALS not HUMANS).sh. (4033465)

8 CLINICAL TRIAL.pt. (506935)

9 exp Clinical Trial/ (849000)

10 (clin$ adj25 trial$).ti,ab. (308227)

11 ((singl$ or doubl$ or trebl$ or tripl$) adj25 (blind$ or mask$)).ti,ab. (146187)

12 PLACEBOS.sh. (34034)

13 placebo$.ti,ab. (174121)

14 random$.ti,ab. (804059)

15 RESEARCH DESIGN.sh. (84544)

16 or/9-15 (1594056)

17 16 not 7 (1478011)

18 17 not 8 (977433)

19 8 or 18 (1484368)

20 exp ORTHODONTICS/ (46224)

21 orthod$.mp. (53863)

22 20 or 21 (61325)

23 (retain$ or retent$).mp. (294935)

24 (fixed$ or removable$ or bonded$ or Essix$ or Hawley$).mp. (221824)

25 22 and 23 and 24 (1152)

26 25 and 19 (174)

## Appendix 2

**Table 6:** Excluded studies with reasons for exclusion (*n* = 36)

Reason for exclusion	Studies
Irrelative outcome to the systematic review	[42–63]
Cross sectional study	[64–67]
Follow-up less than 6 months	[68–70]
Subjects did not undergo orthodontic treatment	[71, 72]
Retrospective study	[73–76]
No control group	[77]
